# A System of Dental Surgery

**Published:** 1859-10

**Authors:** 


### REVIEW.

### ARTICLE IX.

*A System of Dental Surgery.* By John Tomes, F. R. S., &c. 16mo. pp. 599. London: John Churchill, 1859.

An author in presenting himself to the world, is expected to be a master, perfect upon the various subjects about which he feels himself called upon to write. Not only perfect as to what is already known in this relation, but he is expected, and reasonably required to have obtained a clearer insight, and a more extended knowledge of his subject, or a better power of description, than can be found in other works upon similar subjects.

It is perfectly just and necessary that this should be exacted from each new aspirant, more especially when he seeks honor, emolument and position from attempts to add to any branch of the curative art, simply because errors are fatal to the public health and injurious to the cause of science, ofttimes ruinous to the student and seeker after truth, acting as weedy incumbrances to earnest and true progress. Besides, we have an abundance of bad works, and in the few that are creditable to their authors and useful to the pursuit, there certainly should be a much higher degree of exactitude in professional authorship, than is commonly admitted among mere writers of pleasurable fiction for general taste.

Certainly, who could overlook or pardon a *book-maker* in our ranks, an individual who simply compiles a work by gleanings from the labors of other men's brains, or who can be excused if incapable of detecting the old theories of his profession, they only being dressed in altered expression of language.

Has our profession a single individual so imbecile as to fail to laugh at a "Practical Treatise" which omits to treat of practice, but is filled with useless or unimportant theories?

Many, by inattention and carelessness may fail to notice error, or misstatement, or even the monstrous habit of borrowing without due credit, but cannot possibly omit noticing that his search after a practical knowledge must be unaccommodated in a work that is all theory and no practice.

On this side of the water, we have arrived at and adopted certain great truths and principles of practice, which we find must underlie all treatises, or acceptable theories ; nor can we countenance for one moment, a doctrine founded upon other than sound, wholesome and correct principles.

We long for additions, we seize upon every extension, and we honestly reward the authors of such, but we are equally earnest in decrying errors, in condemning the efforts of any one who merely possesses the fever for writing, the facility of copying, the art of the *word-milliner,* without the least genius to offer new or useful ideas or principles, who only can embody old ideas in new forms, who gives an expensive book to the world, with only a loud sounding title, the matter of which is either wholly borrowed, or most lamentably fails to teach what it proposes to do.

Imagine a Wilson's Anatomy which omitted to describe the brain or head, or any part of the human system, or see it as it is, a very perfect system of its science, with a new title page, the subject differently divided, a few theories thrown in here and there, to prevent legal troubles, and you find Wilson is not the man who has taught you anatomy, but Tom Jones or John Smith. Yet you reason, Wilson's treatise existed when this John Smith was in his cradle, and you finally resolve yourself into perfect astonishment at the unblushing effrontery and boldness of

John Smith, and there the matter might end were it not for some one, who without occupation, amuses himself by showing the world that this same John Smith has many imitators, and that a certain new book, called in its advertisement, a *Manual of Dental Surgery,* is like an old furnished house with a new occupant. Its only claim to the *manual* being that it has laid its hands upon every acceptable theory within its reach, that these hands reach a long way, having imported many of its useful facts across seas, and with the law of squatter sovereignty, claimed the originality of much that is old but distant in its origin.

We pleasantly venture to say that any one could write a book, such a one at least as this we are about to consider, but we simply ask who of the liberal and intelligent would have written this book? He may tell you in his preface, that he has only made an "attempt" to produce a book; this is well; but he states it to be a "strictly practical work," following this statement by 255 pages of hypotheses "upon teething, calling this mass of confused matter in a small book, his view of a subject" in a limited degree only. When will the subject be inflicted upon the English in extenso?

Mr. Tomes, fearful that his readers who may not be well read in the profession, should imagine that his practical teachings may not be his own, states that the "mere reprint of the methods of others," would, as they have been published "be but a work of supererogation," and that as it is of practical details, he teaches, therefore that they must "necessarily be his own." Then follows a very quiet and modest acknowledgment of obligations to several dentists of note, mentioning a few names, who may have loaned him for a short time a carious tooth, or a curious one, or a plaster model.

He candidly states that his *exact* quotations of words are limited, and to a monstrous fault his accredited indebtedness is also limited. We do not find fault with Mr. Tomes' apology for the length of time this book has been promised to the public, for it must have employed much time to have perused and made his own, by deep research, the amount of theory now in reach of every student, but we do regret that he had not taken many more years to have digested the thoughts of others before producing them as his own.

The handsome acknowledgment made to Mr. Bagg, is quite due, for the cuts are unequalled in any work extant, and will no doubt result in this gentleman bagging the greatest merit of the book.

One great fault of this writer is his remarkable effort to use obsolete words, or those not in common use, rejecting those that are familiar and well understood by every dentist, as may be instanced in the word "*dilaceration*," which properly means *twisted* or *tearing in two*, the author using it to express a malposition of the crown of a tooth upon its root from some abnormal pressure during its development. Also in the word "*pronounced*," meaning developed, and others of like character, calling loudly for too much indulgence for a simple novice; indeed one is led to infer that this author stands upon some favored ground, with a priestly authority to use new terms and applications which to good taste and proper modesty, is affected, arbitrary, and pedantic.

We should probably have omitted to notice the foregoing error of style, being of minor import to those of a more grave character, which we shall endeavor to show before leaving this little volume, had we not found that with this high drawn use of singular words, he also indulges in the use of those terms which are equally as vulgar and loose; for instance, a cavity from decay is frequently called a "*hole;*" "saucer shaped," for a shallow cavity with a large orifice. Fangs and roots are sometimes called "stumps," "*cropping*," meaning extending or running out, a term used only by miners; why it is dragged from its proper place is difficult to determine, especially as it is an inelegant term for a delicate subject.

We cannot dwell longer upon these smaller faults, although they are abundant, yet as less important than the faults of doctrine found in this *"practical work," necessarily based upon the practice of the author.*

Before entering into details we think a most important error has been committed by the author, in not properly distinguishing the difference between the anatomy and the physiology of the maxilla and their various appendages and integuments. He intermingles them in such a manner that to understand his idea of a part of his subject, you must digest his whole book and labor for conclusions. Physiology is understood to embrace the doctrine of vital phenomena, that is, in his subject it means the object and means of the circulation, nutrition, sensation, absorption, generation and formation. Anatomy, the peculiar relation of parts; this author divides nothing, discriminates nothing of these two indispensable separations of the subject, but in almost every paragraph gives you a little of one and of the other, to the utter confusion of both, without the reader is a master in all departments of science, and able to make this division for the author, so necessary for a a full understanding of the subjects. In the pursuit of any branch of the curative art, the division of the various sciences pertaining thereto, such as physiology, anatomy, pathology, etiology, &c., are as necessary to its correct study, as are the parts of speech in the study of language, their proper arrangement quite as indispensable, for they underlie the elementary principles, enabling us to contemplate and understand the laws of life, health and disease. To jumble them together is a most decided retrogression, productive of confusion, or at least reaching only solitary facts which can establish only doubtful or exceptionable conclusions. To lay down as law, the condition of a part from one or two examples, is judging by too limited a standard, and we reasonably require to establish a law in anatomy or physiology, a great many specimens of particular parts at each *precise* age, for the constructive growth and condition, vary, as do the features of the human family, justifying laws only by generalizations from an immense number of cases, and after intense and continued observation.

This treatise strikes one as ascending from particulars to universals, becoming a metaphysical inquiry upon questions of common interest, and with its chief characteristics singularly headed Teething, whilst embracing as many distinct subjects as would be embodied in a treatise upon the vital functions of the body under the improper title of digestion.

Had the author taken up one branch of his subject in clear distinction from its kindred ones, and pursued it with a deep earnestness as to its limits, and then another branch, and so on until all were separately treated, he could then have theorised to some more useful end, and his views would have probably been more easily understood. Suffice it here to say, that this division of the subject in our estimation is indispensable to continuous, accurate and communicable results, which Mr. Tomes has entirely omitted.

Had the writer made this proper and general division of his subject, and not striven to disguise the unquestionable use of the theories of others by a change of arrangement, or a variation in the order of matter, he never would have ran into the error of following Mr. Kölliker in stating "that in whatever direction the jaw has increased, the increase has been produced by additions to the external surface." He then states, "that there are no indications of interstitial growth within, and throughout the substance of the bone," after the age of nine months. These remarks in the physiological formation of bone in the inferior maxilla are thrown in when considering the anatomical relationship of the forming teeth and jaw of a child of nine months. He afterwards cites a case of elongation of the front inferior maxilla as occurring in a hospital, but which is only perfectly described in the American Journal by Dr. S. P. Hullihen, and subsequently in the Dictionary of Dental Science. Several cuts accompany the article, the case and all attending circumstances of which I was personally familiar with, having known the patient before and after the operation performed by Dr. Hullihen. In this case we have unquestionable proof of interstitial growth, and which constitutes the subject of a lecture in the course delivered by Dr. Harris, in the Baltimore College, and which cannot safely be attacked, at least as far as this power is concerned in the inferior maxilla. In the above case, the wonderful deformity occurred by the traction from the integuments attached to the front part of the inferior maxillary, and not at the alveolus, the distortion occurring simply in obedience to the displaced bone upon which it was formed, so that by his own reasoning, he is either guilty of gross ignorance, or a short-sighted misstatement. For elongation could not have taken place without the whole front portion of the jaw moving, and of course its elongation required interstitial increase.

We shall now proceed in a regular but limited manner, to point out the various defects in this book, and in this order we come to consider why an author should pretend to ignore the terms in common use, which are abundantly sufficient for all descriptive purposes. This will be found upon page 38, where he introduces, as if for the first time, the terms *anterior*, *posterior*, *labial*, *lingual*, and not satisfied with the excellent one approximal, as in universal use, he does introduce two less common words, "*mesial*" and "*distal*," and appears to claim originality of the whole, as he offers as excuse for so introducing them, his effort to be "*intelligible*," and to avoid "*confusion*." Why this has been done, we confess ourselves at some loss to conclude, as no other author has experienced any difficulty of this kind, and we have some quite intelligible in our young land, as also in the old world, who have been our teachers, as well as Mr. Tomes, judging from some few good points in his book with which we have been familiar for many years. Had Mr. Tomes ever been a founder of any new theory creditable to himself and useful to his profession, this might be pardonable or even acceptable. A veteran who has generously devoted his life's labor for the good of others, claims much at our hands, but when a young aspirant liberally takes, and returns us a nomenclature not more expressive than the one previously in use, we are forced to remind him of the disproportionate compensation likely to be obtained by such pedantic effort.

We do not think the views advanced in relation to difficult dentition are supported by fact or reason—mere statement of opinions is of very little advantage to our practice, and in this case one is boldly advanced with but little support.

To reason that difficult dentition is always attended with constitutional disturbances, and therefore, which may be the primary, a matter of doubt is a most assailable conclusion, because such a condition is always ushered in by many local symptoms impossible for an intelligent physician to misunderstand, the constitutional effects having been preceded by the local symptoms; for instance, a child is always more or less fretful for some time previous to the difficult eruption of a tooth, profuse discharges of saliva from the mouth, and signs of great nervous disturbance, and in the nineteen cases out of twenty, the part surrounding the advancing tooth shows more or less severe inflammation. How often does it not happen that a child is seized with convulsions during the period of eruption, and relief following at once the incision of the gums, the nervous disturbance ceases, the tooth appears, the patient recovered; again attacked when the next tooth is about to appear, again relieved, and this state of health lasting throughout the period of teething. Such instances are very common, and occurring in children who may certainly be considered ordinarily healthy, who previous and after this period enjoy all the advantages of health. Again, we do not find healthy children always erupting their teeth at a certain time, and in a certain order, for it will be found that precision in this respect is the exception and not the rule; differing in time of eruption, many months, and sometimes several years, not only in the same family but in the same mouth the greatest irregularity will be noticed, as regards time, place and position, and yet unattended by any indications of impaired constitution, but on the contrary, perfectly healthy.

We cannot but be amused with the modest deference of opinion from Mr. Tomes to the old nurses spoken of in p. 53; he says, "the old nurses tell you that the teeth were breeding in the first attack, and in the second cutting the gums;" *a more probable explanation* is, that in the one case they were passing through the alveolar openings, in the other making their way through the gums. It is pleasing to find him rushing to the rescue of old and antiquated ideas, for when thus supported, they are quite harmless and innocent.

In fig. 23, a case is shown where the front alveoli is wanting in all the teeth in sight. He says, with much pertinence, "surely the unusual condition of the bone of the alveolar processes must have been attended with local indications of disorder."

Then follows Dr. West's opinion, to the effect that such cases are "seldom attended with local irritation, or any increase of the pre-existing constitutional disturbances." Mr. Tomes forgets his own convictions to the contrary, and then advances a theory to account for Dr. West's opinion, so that the reader is freely allowed to wander with either in a state of bewilderment at such conflictions of opinion. Indeed, this subjeet is treated so contradictory, and with such an evident want of knowledge and experience, that we are truly surprised he did not remain silent about this, the most important branch of dental surgery, as he has done about the mechanical department of our art. We do not admit, by any means, the charge of a too great prevailing ignorance upon this subject by dentists, for we have well written treatises, founded upon experience and practice, which are used in our schools, forming the base of a sound education in this particular branch of our profession.

Under the head of shedding of the temporary teeth, we have the erroneous doctrine of absorption again ushered in, but with equally novel and erroneous theories and statements; thus he says absorption may commence on any part of the root of a tooth, or at several simultaneously, not uncommonly on the labial surface of the root; this would probably be the case did an adventitious absorptive process attack roots, but as far as our knowledge extends, no such case as labial destruction has ever been seen, or at least recorded by others.

The destruction of fangs, as a rule, first attacks their apices, varying only as the relative position of the tooth brings it in contact with the "carneous tubercle," which may be at times, from physical causes, out of its true position, that is, superior to the advancing permanent tooth, and in contact with the fang of the temporary. Bourdet first proposed the true *modus operandi;* not, as Mr. Tomes would lead one to infer by an absorbing apparel, but by the before mentioned tubercle.

We think the author is quite correct in saying that this process attacking the enamel of teeth has hitherto escaped observation, nor can we see how he has reasoned to the opinion that the absorption of ordinary bone is a similar process, for the enamel containing from 1 to 2 per cent. of animal matter, is totally incapable of being absorbed, whilst ordinary bone, containing from 30 to 40, is only with difficulty absorbed, the one surrounded and interspersed with vessels carrying the elements of destructive and recuperative powers, the other isolated entirely in some cases from such agents, are differences too great to bring about anything approximating to equality. His theory is not by any course of reasoning, tenable, more particularly the statement that the absorbing process alternates with the formative one, at times. This is also an unobserved circumstance.

That nature should instigate a process of waste one day and of repair the next, in a part where such function is entirely unnecessary, is a discovery becoming this book, and well worthy remembrance, for our impressions are, that nature never provides a means without a purpose. We regret here, upon general principles, and in the entire absence of all proof to disagree with Mr. Tomes in this singular alternation of *waste* and *repair* in the fangs of deciduous teeth.

Upon the subject of irregularities we find many sensible, but old positions, and perhaps less objectionable ones than is common to most parts of this treatise. The use of bone as used in regulating, is a common error in English practice.

We find the objectionable features of this manual so numerous that we are compelled to pass many unnoticed, and shall only be able, from the length of this article, to touch upon the most important defects, and these as briefly as possible.

Much to our astonishment, we find on pages 334 and 5, a clumsy apology for bad operations, because a decay "may appear in some other part of a tooth than the cavity filled," is no reason to argue against the received fact, that a tooth well filled is in that point positively preserved. Caries in some other part of a tooth than the portion filled has no more to do with the filling which may have been inserted, than the decomposition of a tooth in some other patient's mouth would have. We rarely have seen a worse excuse for defective operations than is here found ; we had reason to hope that in this department some light might have been offered. Indeed, under the head of "treatment of caries, and substances generally used," we are compelled to object to the mass of matter jumbled together, in a few distinct points only, for want of time.

The preparation of a tooth for filling, is the most important part of the whole operation. Even a bad filling will often do well, if the tooth has been well prepared for filling, without which the best plug will fail; this is almost entirely omitted. About the removal of decay, the treatise in itself is deficient, its only good points are those every recent writer has made common and universal property. Even the shaping of the cavity is a point not well discussed, and its advocacy of "materials" perfectly untenable, for it strongly recommends the use of *cements and amalgams*, attributing bad effects to preparations in which copper is a constituent, which truly and only belong to preparations containing mercury, a component of all amalgams. It infers that the errors of amalgam fillings are attributable to the bad finish generally given to them. As to justifying their use because better metals fail to do what can be done with them is to directly contradict the position and oft repeated assertion of good operators, who state their capability to secure with gold, the preservation of any tooth, the condition of which justifies an attempt to preserve it. The credit due to the United States for introducing the use of soft fillings for temporary purposes is overlooked, recommending even for their use, amalgams as often preferable, when we know that the peculiar sensibility of some teeth will not admit of a metallic substance.

The little that is said about the use of instruments in filling, impresses a good operator with the belief that the author must be clumsy in their use, for no good operator ever requires the use of two instruments at once in the proper introduction of gold into a cavity, or for its treatment when so introduced. This must arise from a bad conception of the manner of using gold in adapting it through various forms to meet all peculiarities in the shape of cavities. This idea, however, is made more evident in reading his conception of the use of cylinders, and in the introduction of the star-shaped method which is finical and labor increasing, never used in America in connection with cylinders or any other preparation of gold that we are aware of. As to the solidity of plugs, it must be a mere waste of time to apply the microscope to ascertain if it were, *de facto*, a perfectly solid body. All desirable wants of solidity can be most certainly obtained, as imperviousness to air and fluid, is all we desire, and if Mr. Tomes' microscope found imperfections, they were not those of the practical man, but the theorist, and we ask, *qui bono?*

For better understanding, we quote one sentence, all can see the loose and peculiar style of treating this particular operation. "The cavity, by *one method* or *another*, having been filled, and the gold allowed to project slightly from the orifice, allowing a slight degree of fulness to the central portion of the plug." How is it possible for the impression of approximal fillings to be taken in wax which is recommended but not explained, we are at a loss to see or to understand the necessity in any case, as it is a matter of no doubt at all to an operator whether a cavity will hold gold or not, any more than it could be of 2 and 2 making 4. Labial fillings which so often prove of great difficulty, are regarded by this author as a "very simple kind." When we think of the almost universal cause, defective formation, and the peculiar form of tooth surface, sloping gradually down into the cavity, rendering the formation of a good one almost impossible, and certainly not enabling us in most cases, to secure exemption to the part from subsequent decay; we are astonished at the difference of opinion that the separating ocean causes.

The use of adhesive plaster is, we confess, quite novel, nor do we see the propriety of its adoption; no trouble is experienced in keeping most teeth dry while filling, simply by the use of small pieces of linen; the idea of using such a material itself is alone disagreeable and troublesome, nor do we see what is to prevent the saliva from flowing over it, in a little time, doing as much mischief as could occur without its use.

One cannot but remark the great similarity of instruments represented in all cases but two, figs. 161 and 162; these we have no ambition to recognize, as we never use such inappropriate shapes, but the others resemble American patterns so exactly, that we should think the author had used ours as models, did he not remain silent upon this head.

It seems, from the absence of all acknowledgment in this book, that we deserve no credit for first introducing, and subsequently perfecting fang filling; this, with many other points, seem to be quite too insignificant to date the authorship of, and this would really be of small moment, did the author show the least indication of liberality or justice, did he lead one to infer the existence of any other writer who had done anything good or of moment, excepting to mix some new cement or meet with some strangely grown tooth, and they having had the good taste to bring it to Mr. Tomes, who has obligingly shown it to the dental public. Perhaps in one or two instances he gives methods of others, but invariably ends by his own, which, if good, is that, we all have been accustomed to use, sometime before Mr. T. was a dentist.

We cannot forbear referring to one gross error found in several parts of this book, viz. that a certain mechanical injury results from the improper use of the tooth-brush: in reply to which, we simply assert, this is absolute nonsense, and venture to say it is quite impossible for the brush, used *without injurious* powders, to do harm in any way to the teeth.

His theory about the conversion of the pulp into secondary dentine is wrong and unphilosophical. The treatment of diseased gums is not such treatment as we pursue in the United States, any more than are his ideas about the sloughing of gums after lancing. No tooth ever falls out without having become obnoxious, and when this takes place, then nature sets up a process to get rid of it. Nor can we see why dentists should not pursue the practice of the surgery of the face, especially that belonging to the jaws. Does Mr. Tomes confess an ignorance upon necrosis of the alveolar processes, that he recommends research in works upon *general surgery* for necrosis of the maxilla?

When we began to review this work, we imagined that it could not extend over a few pages, but since a more careful reading, we are satisfied that it would require a whole number of the Journal to mention and correct all the errors and fallacies it contains. We, therefore, close the task, feeling that a necessary and desirable review of the whole cannot be accomplished in a single paper, and that the influence of such a work here would hardly justify the expenditure of the time it would cost. The evident attempt to sneer at the doctrine and practice of others, the bitter, yet hidden, remarks occasionally found in this book is an introduction of a new feature into manuals, not to be encouraged. Nor can we conclude that the author has raised himself one particle in any liberal man's estimation; for an author's first duty is justice to others. The reader of this work will be struck with the vanity and pedantry of the writer.

