# Errata

**Published:** 1859-10

**Authors:** 


**On page 550, 8th line from top, read *maxillæ*, for maxilla.**

**On page 554, 19th line from top, read, after front, *of the.***

